# Supplementary material for: Design and validation of a foldable and photovoltaic wide-field epiretinal prosthesis
Source: Nat Commun. 2018 Mar 8;9:992. doi: 10.1038/s41467-018-03386-7 (PMC5843635; doi:10.1038/s41467-018-03386-7)
Supplement: Supplementary file 1 — Supplementary Information [file 41467_2018_3386_MOESM1_ESM.pdf]

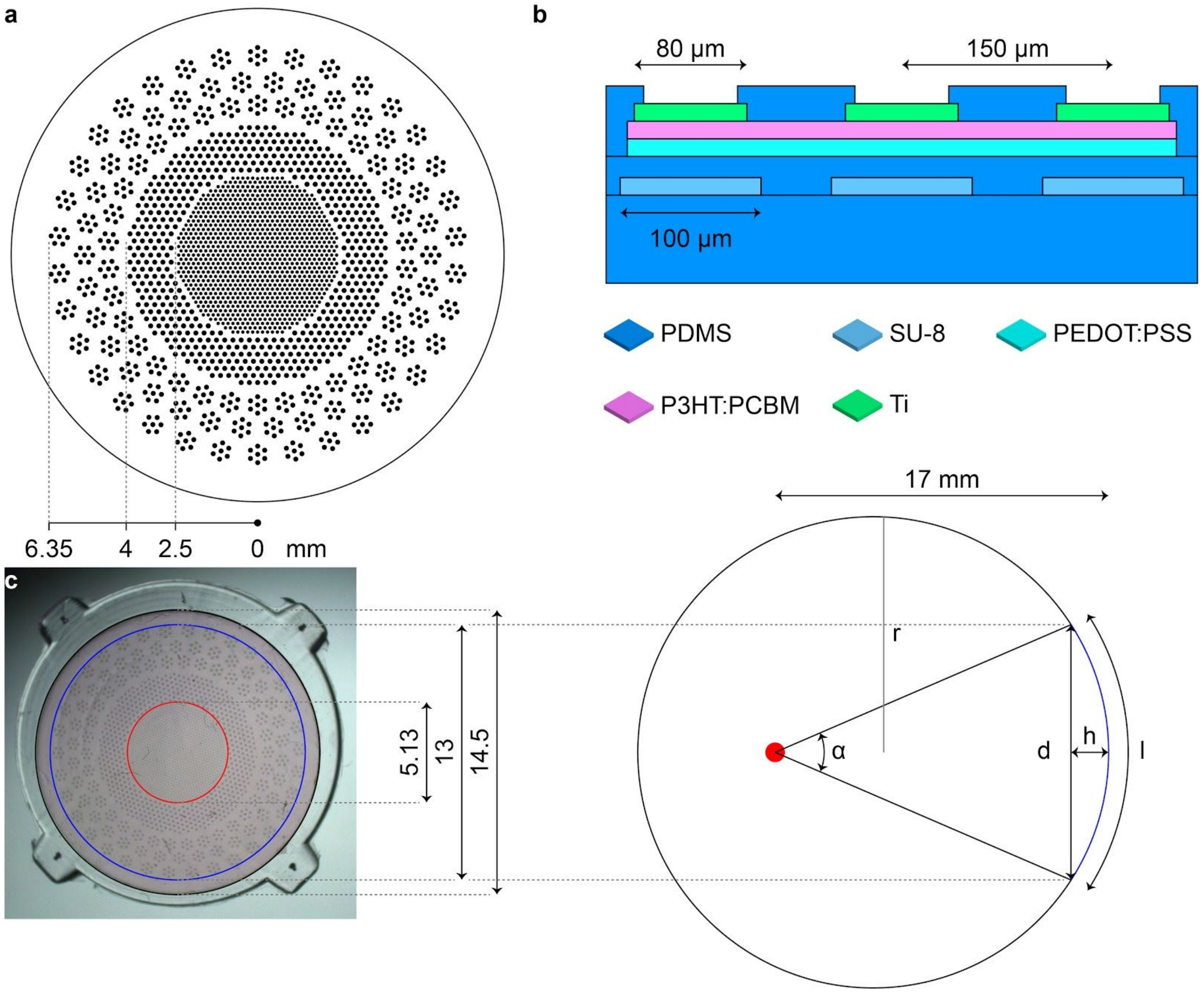

Supplementary Figure 1 | Design of POLYRETINA. **a**, Distribution of the photovoltaic pixels. **b**, Cross section of the PDMS-photovoltaic interface, including: PDMS (50 µm), a second layer of PDMS (10 µm) embedding SU-8 rigid platforms (6 µm), a layer of PEDOT:PSS (50 nm), a layer of P3HT:PCBM (100 nm), titanium cathodes (150 nm), and a final layer of PDMS (4 µm). Thicknesses are not in scale. **c**, Picture of the prosthesis after bonding. Due to the radial elongation, the central area (red) is slightly stretched from 5 to 5.13 mm, while the active area (blue) is increased from 12.7 to 13 mm. On the right, the visual angle is calculated. The human eye is modelled as a sphere of 12 mm radius (r). The blue arc corresponds to the active area, where d is the chord length of 13 mm, l is the arc length of 13.738 mm, and h is the height of 1.9128 mm. The nodal point is represented in red, with a distance of 17 mm. Under these conditions, the area covered by the active area can be calculated as $S = 2\pi rh = 144.2217\ mm^2$, and it corresponds to a visual angle α of 46.3° (or 808.12 mrad).

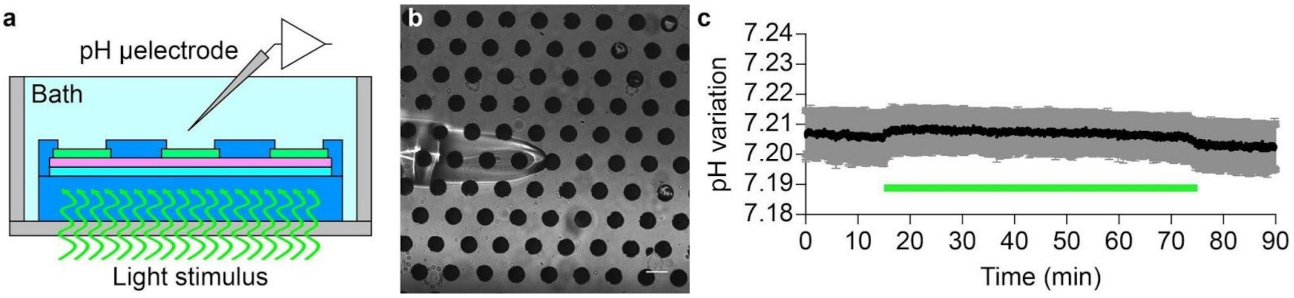

Supplementary Figure 2 | pH measurements upon prosthetic stimulation. **a**, Sketch of the recording set-up. **b**, Picture of the pH microelectrode located on top of the PDMS-photovoltaic interface. The scale bar is 100 µm. **c**, Mean (± s.d., N = 3 devices) pH measurements upon 1 hr of full field pulsed illumination (10 ms, 20 Hz, 3.4 mW mm^{-2}, 560 nm, illumination spot 2.2 mm).

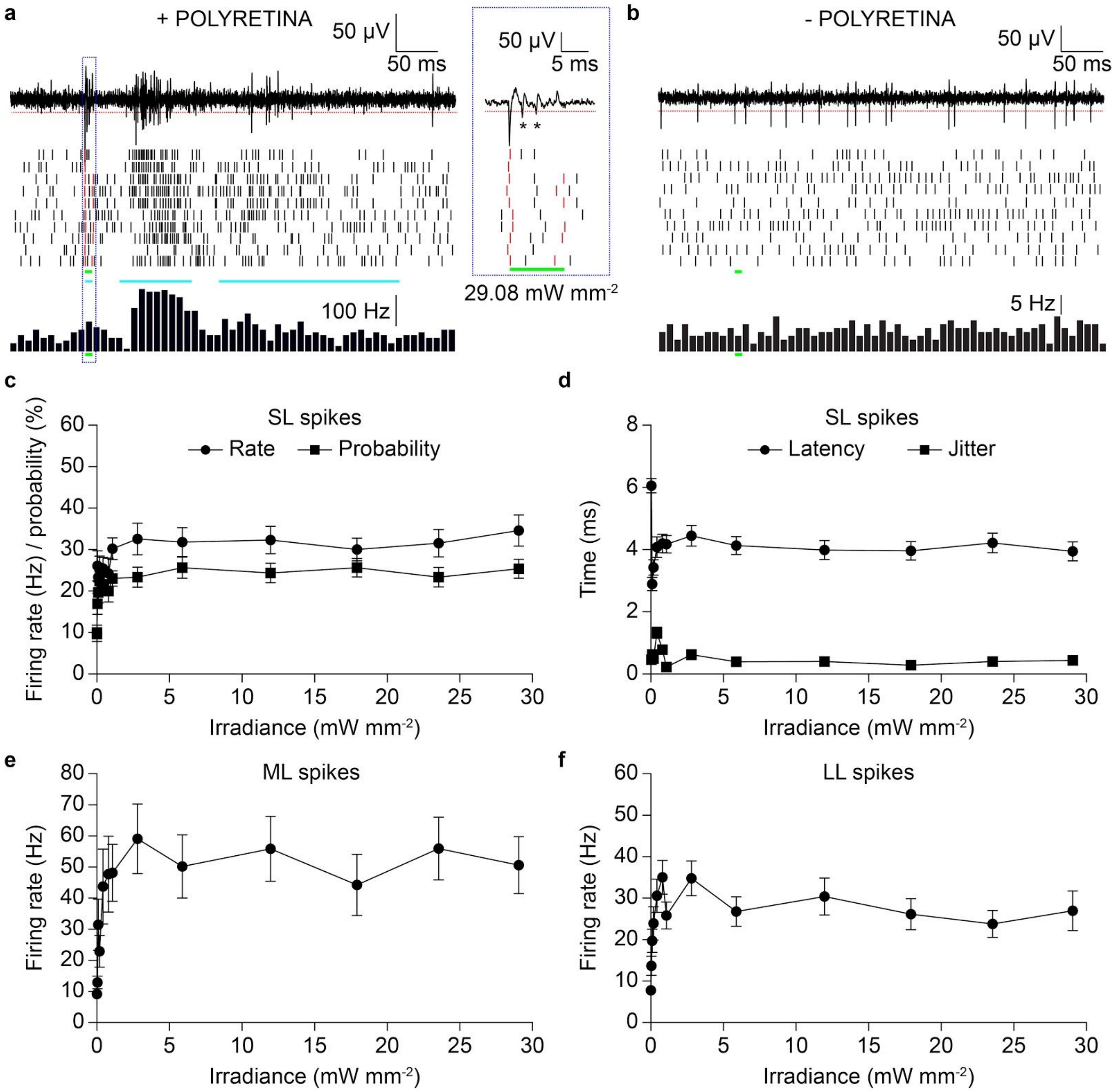

Supplementary Figure 3 | Evaluation *ex-vivo* with retinal explants. **a**, The top panel shows a representative single-sweep recording from a retinal ganglion cell over the PDMS-photovoltaic interface upon maximal illumination (10 ms, 29.08 mW mm^{-2}). The red dotted line is the threshold set for spike detection. The middle panel shows the raster plot based on the over-threshold events detected and classified as spikes upon 10 consecutive sweeps in the same cell. The green bars represent the light illumination. The cyan bars represent the regions where SL, ML, and LL spikes have been identified. The red bars correspond to the detection of the stimulation artefacts at the onset and offset of illumination. Artefacts have been excluded in subsequent analysis. The bottom panel shows the PSTH of the cell computed over 10 consecutive sweeps.

The blue box shows an enlarged view of the light onset. The asterisks (*) indicate the over-threshold events detected and classified as spikes. **b**, Example from a retinal ganglion cell over bare PDMS. **c**, Mean (± s.e.m.) firing rate (●) and firing probability (■) of SL spikes, computed across all the recorded cells (*n* = 39) on the PDMS-photovoltaic interface. For each cell, the probability has been defined as the percentage of sweeps with at least a SL spike over the 10 consecutive trials. **d**, Mean (± s.e.m.) latency (●) and jitter (■) of the first spike occurring in the 10 ms window after the light onset, computed across all the recorded cells (*n* = 39, 10 sweeps each) on the PDMS-photovoltaic interface. **e,f** Mean (± s.e.m.) firing rate of medium (**e**) and long (**f**) latency spikes, computed across all the recorded cells (*n* = 39, 10 sweeps each) on the PDMS-photovoltaic interface.

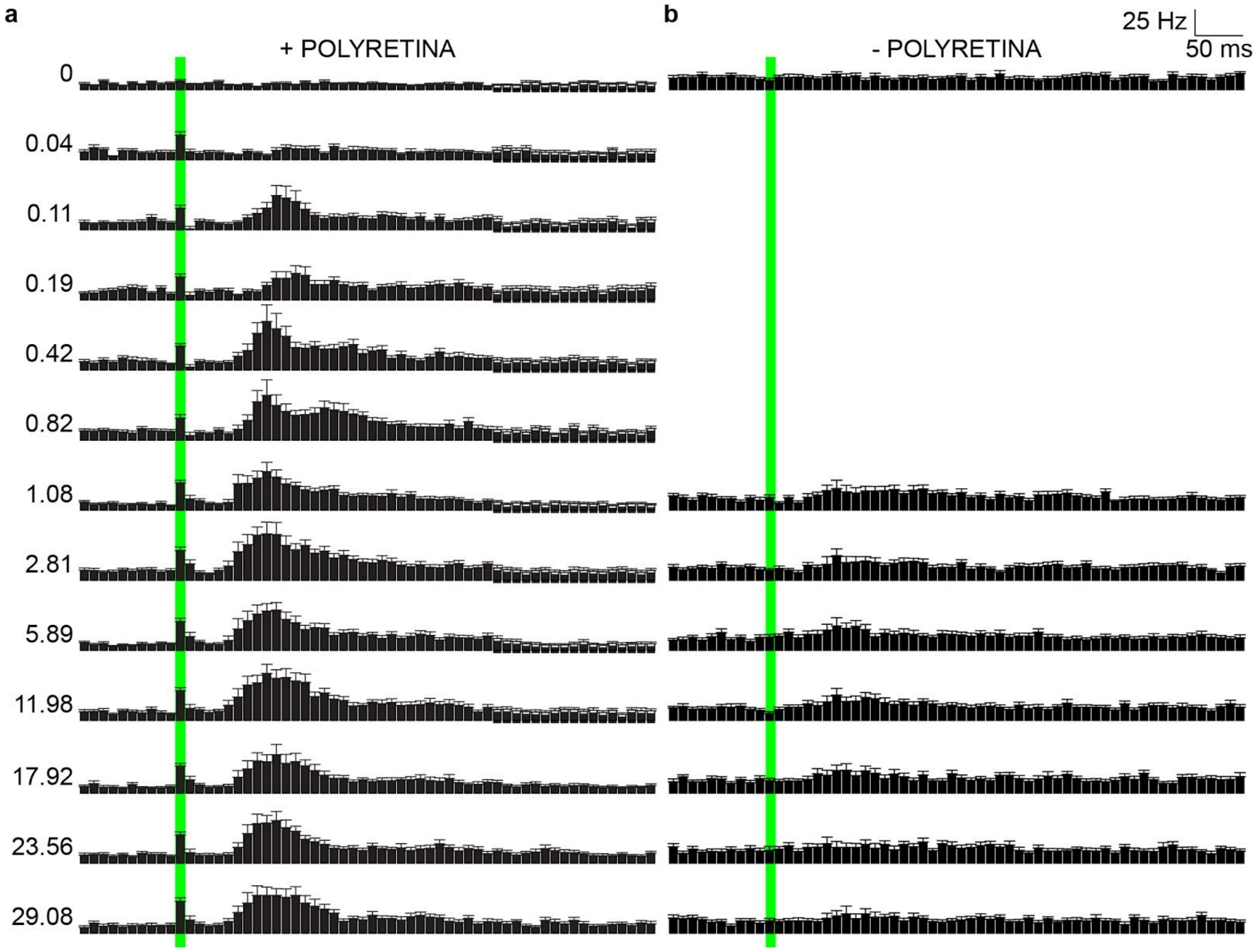

Supplementary Figure 4 | Recording of *Rd10* retinas *ex-vivo*. PSTHs (bin 10 ms, mean ± s.e.m.) obtained from n = 39 and n = 34 retinal ganglion cells, respectively for the PDMS-photovoltaic interface (**a**) and the bare PDMS substrate (**b**). Each row corresponds to a different light intensity expressed on the left in mW mm^{-2}. Green bars represent the light pulses. On bare PDMS substrate, cells have been tested only for the high range of irradiance.

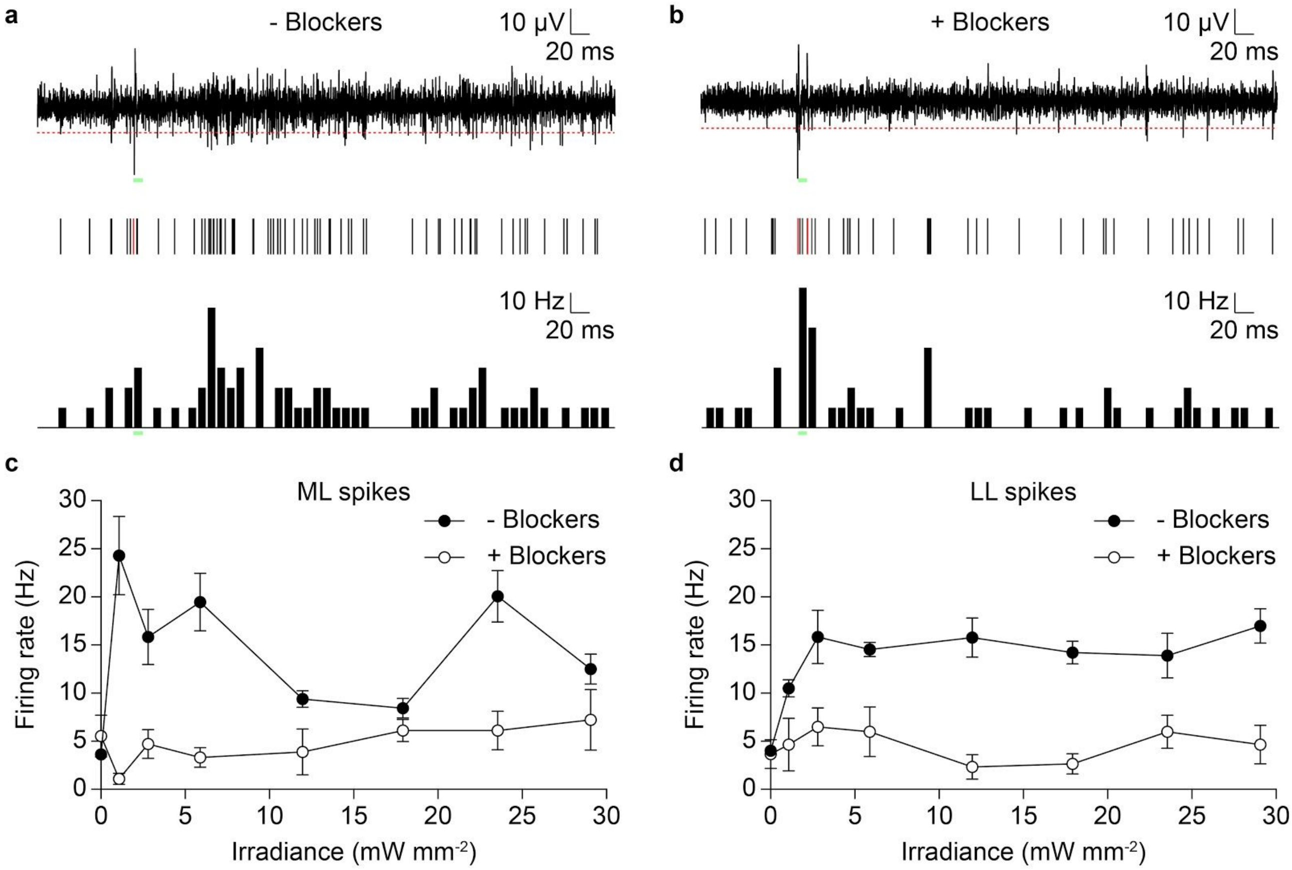

Supplementary Figure 5 | Pharmacological blockage of network activity. **a**, The top panel shows a representative single-sweep recording from a retinal ganglion cell over the PDMS-photovoltaic interface upon illumination (10 ms, 23.56 mW mm^{-2}). The red dotted line is the threshold set for spike detection. The green bars represent the light pulse. The middle panel shows the raster plot based on the over-threshold events detected and classified as spikes upon 10 consecutive sweeps (overlay) in the same cell. The red bars correspond to the detection of the stimulation artefacts at the onset and offset of illumination. Artefacts have been excluded. The bottom panel is the PSTH (bin 10 ms) of the cell computed over 10 consecutive sweeps. **b**, Response upon illumination (10 ms, 23.56 mW mm^{-2}) of the same retinal ganglion cell in **a** , after inclusion of synaptic blockers. Panels **c**/**d** respectively show the mean (± s.e.m.) firing rate of medium/long latency spikes, computed across all the recorded cells (*n* = 6, 10 sweeps each) on the PDMS-photovoltaic interface before (●) and after (○) the inclusion of synaptic blockers.

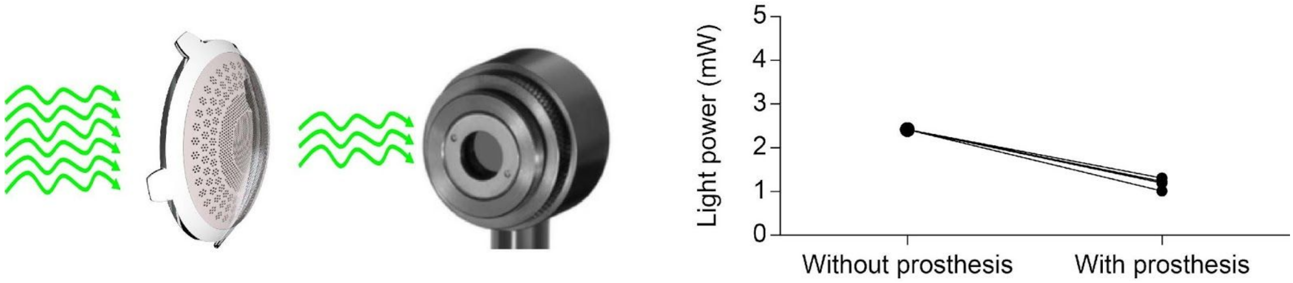

Supplementary Figure 6 | Optical absorption. Light absorbance/transmittance of POLYRETINA has been evaluated by using a green LED (565 nm, 2.42 mW). Light has been measured with a power meter (PD300-R Juno, Ophir Optronics Solutions Ltd.). The retinal prostheses (*N* = 4) have been inserted in the light path and the light power has been compared with respect of the condition without the prostheses.

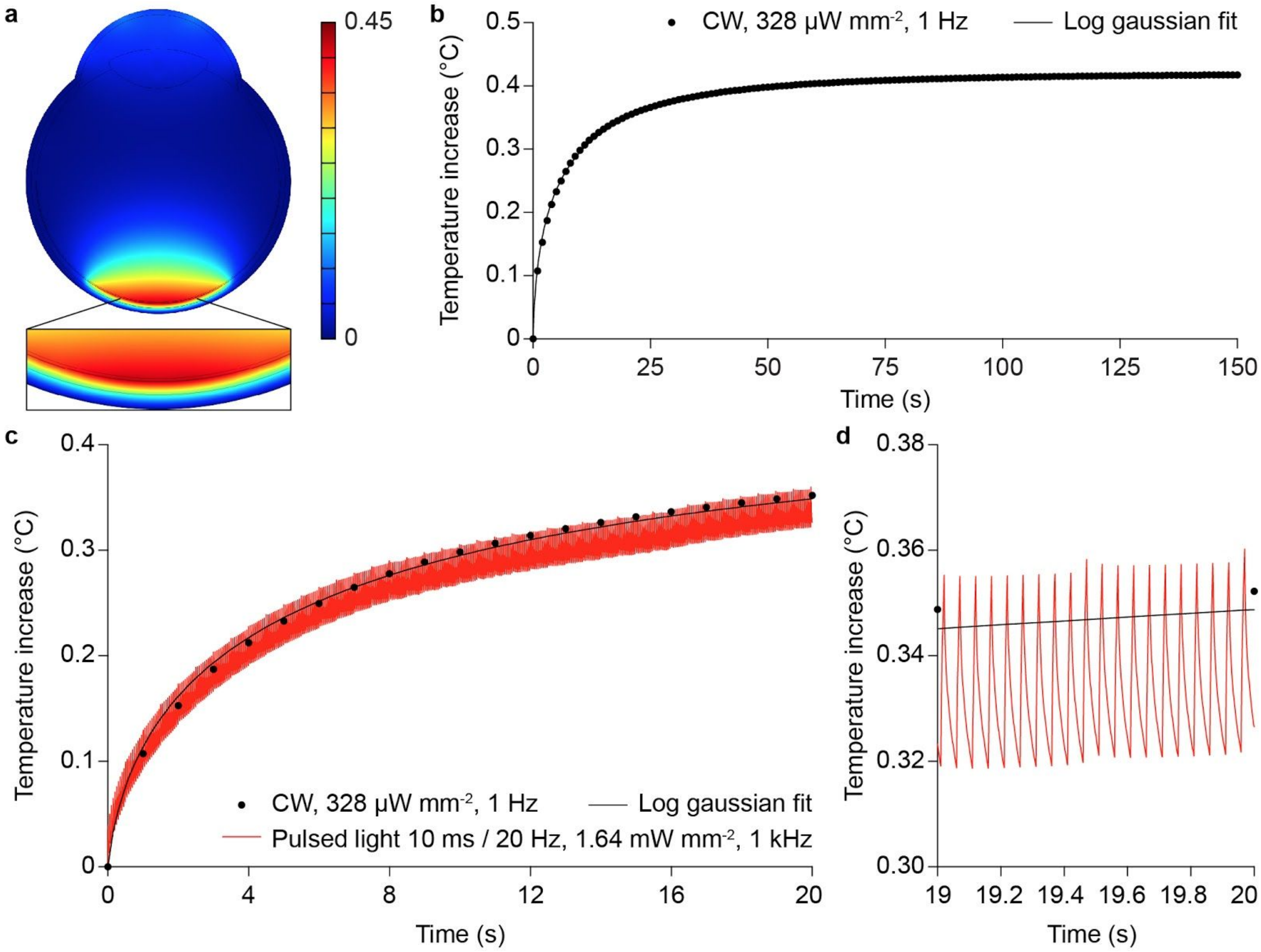

Supplementary Figure 7 | FEA simulation of thermal effects. **a**, Temperature increase in the modelled eye after 150 s of continuous illumination (CW, 560 nm, 328 μW/mm^2). The insert shows a larger view of the modelled retina. **b**, Time course of the temperature increase in the modelled retina during 150 s of continuous illumination (CW, 560 nm, 328 μW/mm^2). The simulation frequency has been set to 1 Hz. The line is the log gaussian fit (R^2 = 0.9958). **c**, Comparison of the temperature time course during continuous illumination at 328 μW/mm^2 (black dots) and pulsed illumination with 10 ms pulses at 20 Hz and 1.64 mW/mm^2 (red line) for 20 s of simulation. The simulation frequency for the pulsed illumination has been set to 1 kHz. **d**, Magnification of the last 1 s of the simulation in **c**.

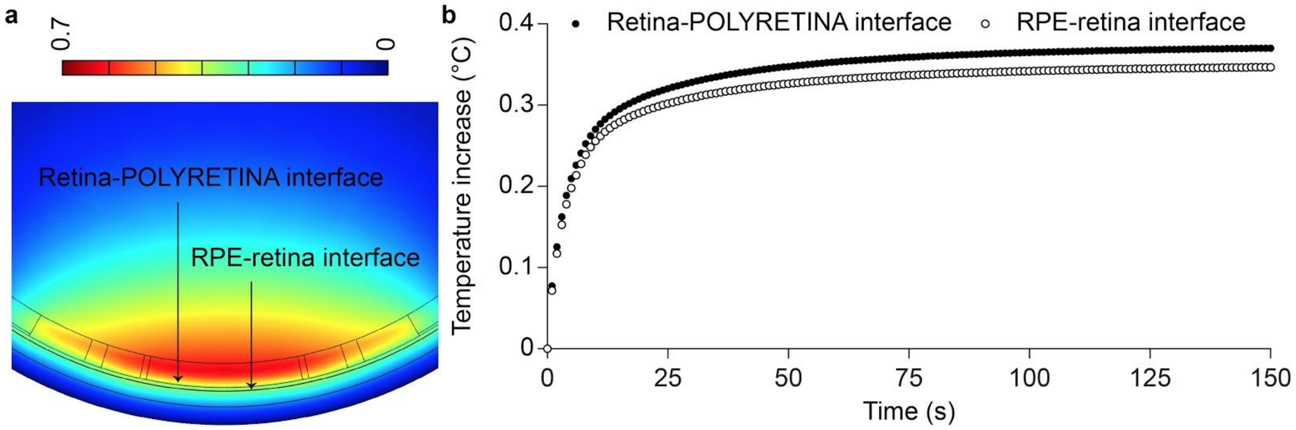

Supplementary Figure 8 | FEA simulation of thermal effects with POLYRETINA. **a**, Enlarged view of the temperature increase in the modelled eye with POLYRETINA after 150 s of continuous illumination (CW, 560 nm, 328 μW mm^{-2}). **b**, Time course of the temperature increase at the RPE-retina (○) and retina-POLYRETINA (●) interfaces during 150 s of continuous illumination (CW, 560 nm, 328 μW mm^{-2}).

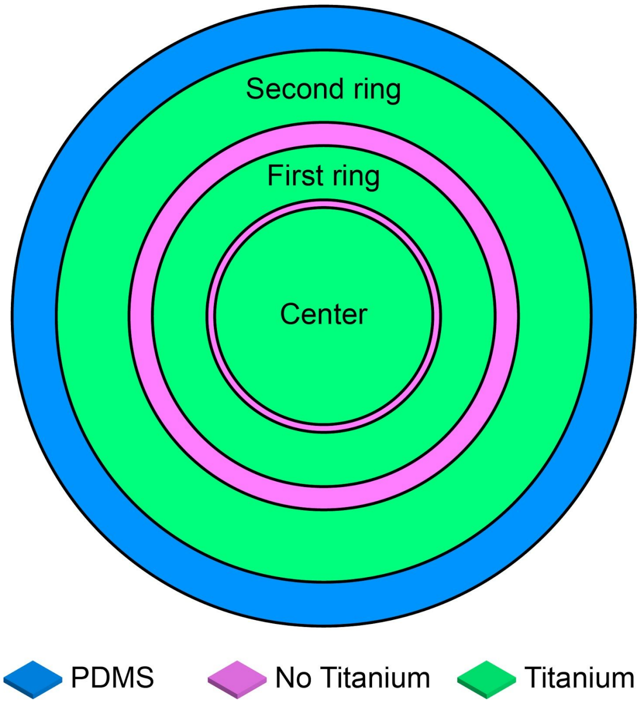

Supplementary Figure 9 | Aggregated model of POLYRETINA. Drawing of the simplified model of POLYRETINA.

Material	Thickness	Heat Capacity	Thermal Conductivity	Density	Absorption (at λ in nm)	Perfusion rate	Self Heat
	μm	J kg^{-1} K^{-1}	W m^{-1} K^{-1}	Kg m^{-3}	1 cm^{-1}	1 s^{-1}	W m^{-3}
**Eye**	ø 24000						
**Aqueous Humor**	3100	3997	0.58	1000	0.00025 (500)	0	0
**Blood**	/	3840	0.53	1050	0	0	0
**Choroid**	430	3840	0.53	1050	150 (500)	0.0091	10000
**Cornea**	500	4178	0.58	1050	0.51 (514.5)	0	0
**Lens**	3600	3000	0.4	1000	0.025 (514.5)	0	0
**Retina**	100	3680	0.565	1000	4 (500)	0	0
**RPE**	10	4178	0.603	1050	1100 (500)	0	0
**Sclera**	500	4178	0.58	1000	5.9 (550)	0	0
**Vitreous Humor**	/	3997	0.595	1050	0.00025 (500)	0	0
**PDMS**	669	1460	0.15	970	3.58 (514.5)	0	0
**PEDOT:PSS**	0.15	1978	0.29	1011	1700 (500)	0	0
**P3HT:PCBM**	0.1	1400	0.2	1100	40000 (530)	0	0
**Titanium**	0.05	5263	6.7	4430	120000 (500)	0	0

Supplementary Table 1 | FEA simulation of thermal effects. Parameters used in the thermal model obtained from Supplementary References 1-14.

Domain	Heat Capacity	Thermal Conductivity	Density	Absorption (at λ in nm)	Fraction of Titanium
	**J kg^{-1} K^{-1}**	**W m^{-1} K^{-1}**	**Kg m^{-3}**	**1 cm^{-1}**	**%**
**Center**	1460.89	0.15	970.07	16.91	27
**First ring**	1460.89	0.15	969.90	12.30	10
**No titanium**	1459.70	0.15	969.80	9.73	0
**Second ring**	1460.89	0.15	969.85	11.09	0.5
**PDMS**	1460.00	0.15	970.00	3.58	0

Supplementary Table 2 | FEA model of POLYRETINA. Parameters used to generate the aggregated model of POLYRETINA used in the thermal simulations. Parameters can be obtained from Supplementary References 1-14.

Supplementary References

1. Hammer, M., Roggan, A., Schweitzer, D. & Müller, G. Optical properties of ocular fundus tissues--an in vitro study using the double-integrating-sphere technique and inverse Monte Carlo simulation. *Phys. Med. Biol.* **40,**963–78 (1995).

2. Johnson & Christy. Optical constants of transition metals: Ti, V, Cr, Mn, Fe, Co, Ni, and Pd. *Phys. Rev. B* **9,**5056–5070 (1974).

3. Narasimhan, A. & Jha, K. Bio-heat transfer simulation of square and circular array of retinal laser irradiation. *Front. Heat Mass Transfer* **2,** (2011).

4. Sardar, D., Yust, B., Barrera, F., Mimun, L. & Tsin, A. Optical absorption and scattering of bovine cornea, lens and retina in the visible region. *Laser Med. Sci.* **24,** 839–847 (2009).

5. Mirnezami, S., Jafarabadi, M. & Abrishami, M. Temperature distribution simulation of the human eye exposed to laser radiation. *J Lasers Medical. Sci.* **4,** 175–81 (2013).

6. Gosalia, K., Weiland, J., Humayun, M. & Lazzi, G. Thermal Elevation in the Human Eye and Head Due to the Operation of a Retinal Prosthesis. *IEEE Trans. Biomed. Eng.* **51,** 1469–1477 (2004).

7. Martino, N. *et al.* Photothermal cellular stimulation in functional bio-polymer interfaces. *Sci Rep* **5,** 8911 (2015).

8. Sarnaik, R., Chen, H., Liu, X. & Cang, J. Genetic disruption of the On visual pathway affects cortical orientation selectivity and contrast sensitivity in mice. *J. Neurophysiol.* **111,**2276–2286 (2014).

9. Liu, J. *et al.* Thermal Conductivity and Elastic Constants of PEDOT:PSS with High Electrical Conductivity. *Macromolecules* **48,** 585–591 (2015).

10. Wang, J. *et al.* Retinal safety of near-infrared lasers in cataract surgery. *J. Biomed. Opt.* **17,** 0950011–09500112 (2012).

11. Brown, J. *et al.* In Vivo Human Choroidal Thickness Measurements: Evidence for Diurnal Fluctuations. *Invest. Ophthalmol. Vis. Sci.* **50,** 5–12 (2009).

12. Stankova, N. E. *et al.* Optical properties of polydimethylsiloxane (PDMS) during nanosecond laser processing. *Appl. Surf. Sci.* **374,** 96–103 (2016).

13. Müllerová, J., Kaiser, M., Nádaždy, V., Šiffalovič, P. & Majková, E. Optical absorption study of P3HT:PCBM blend photo-oxidation for bulk heterojunction solar cells. *Sol Energy* **134,** 294–301 (2016).

14. Mark J. Polymer Data Handbook. Oxford Univ Press, New York (1999)

Supplementary Certificate

The original document with official signatures can be shared upon request.

Medistri.SA
a must in medical chain

SWISS ACCREDITATION sas.admin.ch STS 0504

Rte de l'Industrie 96
Case postale 115
1564 Domdidier
Switzerland

Tel : +41 (0) 26 676 90 80
Fax: +41 (0) 26 676 90 85
TVA: 645 904

www.medistri.com
contact@medistri.com

Routine analysis / *Résultat d'analyse de routine*

Biological evaluation of medical devices: in vitro cytotoxicity test

Evaluation biologique des dispositifs médicaux: test de cytotoxicité in vitro

General Information

Customer name	**E.P.F.L.**
Customer address	**Chemin des Mines 9**<br>**CH-1202 Genève**
Date of reception of the product	**18th April 2017**
Product identification (name and reference)	**Sample ID:** 1 — **Name of the Product:** POLYRETINA — **Manufacturing Batch Reference:** ALPHA.01 / ALPHA.02
Customer's reference (Delivery note #; order #...)	**EPFL**
Product surface area (cm²)	**Sample ID:** 1 — **Product surface area (cm²) / piece:** 1.77 cm²
Packaging conditions	☐ Under vaccum packaging ☒ Standard packaging
Sterilization conditions	☒Sterile :<br>☒ Medistri SA :<br>Ste batch # **17-0375-1 / 17-0377-1** ☐ Other<br>☐Non sterile
Quantity of samples	1 pool of 2

Method

The study was conducted according to the requirement of SN ISO 10993-5 : Biological Evaluation of Medical Devices, *in vitro* cytotoxicity test, SN ISO 10993-12: Test article preparation and reference materials, USP 35-NF30 (87): Biological Reactivity test, *in vitro* and Medistri internal procedure WI 47 and WI 56. All current versions

Test method	☐ Direct contact ☐ Agar diffusion ☒ Test on extract
Tested surface area (cm²)	**Sample ID:** 1 — **Product surface area (cm²) / piece:** 1.77 cm² — **Number of pieces tested:** 2 — **Total surface area (cm²) tested:** 3.54 cm²
Ratio of the product to extraction vehicle (cm²/ml)	3 cm²/ml
Extraction vehicle	☒ EMEM medium — Batch #: **RNBF3691**<br>☐ other (specify): N.A. — Batch #: **n.a.**
Medium supplemented with	☒ Fetal bovine serum — Batch #: **1110D**<br>☒ Penicillin-streptomycin — Batch #: **A2213**<br>☒ Amphotericin B — Batch #: **1407D**<br>☒ L-glutamine — Batch #: **0951C**
Extraction conditions	☒ (24±2) h at (37 ±1)°C ☐ other (specify): N.A:
Test system	L929 cell line (ECACC n°88102702). The murine fibroblastic L929 cells are recommended by ISO 10993-5.
Incubation conditions for cells	37 ±1°C and 5 ±1% CO_2
Date of start	18th April 2017
Date of end	20th April 2017

Medistri.SA

a must in medical chain

SWISS ACCREDITATION sas.admin.ch STS 0504

Rte de l'Industrie 96
Case postale 115
1564 Domdidier
Switzerland

Tel : +41 (0) 26 676 90 80
Fax: +41 (0) 26 676 90 85
TVA: 645 904

www.medistri.com
contact@medistri.com

Description

The product or its extract was added on triplicate culture wells containing a sub-confluent L929 cell monolayer. The test sample and the control wells were incubated at 37 ± 1°C in 5 ± 1% CO_2 for a minimum of 24 hours.

Following incubation, the cell cultures were examined for qualitative and/or quantitative cytotoxic evaluation.
For qualitative evaluation, cells were stained and examined macroscopically for cell decolorization around the portion of the product. The cultures were then examined microscopically to verify any decolorized zones and to determine cell morphology in proximity to and beneath the tests and controls.

For quantitative evaluation, 50 µl/well of XTT reagent was added to the cells then incubated at 37 ± 1°C in 5 ± 1% CO_2 for further 3-5h. An aliquot of 100 µl was then transferred from each well into the corresponding wells of a new plate and the optical density (OD) was measured at 450nm.

Results_96 well microplate

Samples ID	*Sample dilution*	% viability** (quantitative evaluation)	Acceptance criteria	Cytotoxic effect
			% viability	
1	1:1	100	If < 70% (compared to negative control), it has a cytotoxic effect	Yes ☐ No ☒
**Samples**	*Sample dilution*	**% viability**** (quantitative evaluation)	**Acceptance criteria**	**Pass/Fail**
			% viability	
Positive control	1:1	0.3	n.a.	Pass ☒ Fail ☐
Negative control	1:1	100	n.a.	Pass ☒ Fail ☐
Blank	1:1	n.a.	n.a.	n.a.

*** Viability max is 100%.*

Conclusion

The device tested is not cytotoxic under the condition of the test.

Approvals

Date : **21st April 2017** [redacted]
Responsible for analysi[redacted]

Date : 24th April 2017
Quality Department : [redacted]
